# Pharmacological correction of excitation/inhibition imbalance in Down syndrome mouse models

**DOI:** 10.3389/fnbeh.2015.00267

**Published:** 2015-10-20

**Authors:** Benoit Souchet, Fayçal Guedj, Zsuza Penke-Verdier, Fabrice Daubigney, Arnaud Duchon, Yann Herault, Jean-Charles Bizot, Nathalie Janel, Nicole Créau, Benoit Delatour, Jean M. Delabar

**Affiliations:** ^1^Université Paris Diderot, Sorbonne Paris Cité, Adaptive Functional Biology, UMR Centre National de la Recherche Scientifique 8251Paris, France; ^2^Tufts Medical Center, Mother Infant Research InstituteBoston, MA, USA; ^3^Université Pierre-et-Marie-Curie Paris, 06 UMR S 1127, Centre National de la Recherche Scientifique UMR 7225, Institut National de la Santé et de la Recherche Médicale, U 1127, Sorbonne Universités, Institut du Cerveau et de la Moelle EpiniereParis, France; ^4^Institut Génétique Biologie Moléculaire Cellulaire, Centre National de la Recherche Scientifique, Institut National de la Santé et de la Recherche Médicale, UMR7104, UMR964Illkirch, France; ^5^Key-ObsOrléans, France

**Keywords:** Down syndrome, DYRK1A, EGCG, GABA pathway, glutamate pathway, excitation/inhibition balance

## Abstract

Cognitive impairment in Down syndrome (DS) has been linked to increased synaptic inhibition. The underlying mechanisms remain unknown, but memory deficits are rescued in DS mouse models by drugs targeting GABA receptors. Similarly, administration of epigallocatechin gallate (EGCG)-containing extracts rescues cognitive phenotypes in Ts65Dn mice, potentially through GABA pathway. Some developmental and cognitive alterations have been traced to increased expression of the serine-threonine kinase *DYRK1A* on Hsa21. To better understand excitation/inhibition balance in DS, we investigated the consequences of long-term (1-month) treatment with EGCG-containing extracts in adult mBACtgDyrk1a mice that overexpress *Dyrk1a*. Administration of POL60 rescued components of GABAergic and glutamatergic pathways in cortex and hippocampus but not cerebellum. An intermediate dose (60 mg/kg) of decaffeinated green tea extract (MGTE) acted on components of both GABAergic and glutamatergic pathways and rescued behavioral deficits as demonstrated on the alternating paradigm, but did not rescue protein level of GABA-synthesizing GAD67. These results indicate that excessive synaptic inhibition in people with DS may be attributable, in large part, to increased *DYRK1A* dosage. Thus, controlling the level of active DYRK1A is a clear issue for DS therapy. This study also defines a panel of synaptic markers for further characterization of DS treatments in murine models.

## Introduction

Down syndrome (DS), occurring in 1 in every 750 live births, encompasses a constellation of features caused by partial or complete trisomy for chromosome 21 (Hsa21). In particular, an altered copy number for segments of Hsa21 containing the dual-specificity tyrosine phosphorylated and regulated kinase 1A (*DYRK1a*) gene can induce morphological defects and cognitive impairments (Delabar et al., 1993; Ronan et al., 2007; van Bon et al., 2011). These defects have been reproduced in a number of different mouse models of DS (Ts1Rhr, Ts65Dn, Ts1Cje, Dp(16)1Yey) as well as mice with altered copy numbers of *Dyrk1a* (hBACtgDyrk1a, hYACtgDyrk1a, mBACtgDyrk1a, *Dyrk1a*+/−). Interestingly, a phenotype rescue experiment crossing Ts65Dn mice, which have three copies of *Dyrk1a*, with mice monosomic for a 33-gene chromosomal segment containing *Dyrk1a* (Ms1Rhr) produced progeny with a normal learning phenotype, indicating that triplication of this 33-gene region is necessary to produce the cognitive deficit (Belichenko et al., 2009). A complete phenotypic assessment of Ts1Rhr mice, trisomic for the 33-gene segment, showed that trisomy of this region is sufficient to produce significant alterations in behavioral tasks such as the open-field, novel object recognition, and T-maze tasks. In Ts65Dn, Ts1Cje, and Ts1Rhr mice, long-term potentiation (LTP) in fascia dentata (FD) could be induced only after blocking GABA(A)-dependent inhibitory neurotransmission. In addition, widespread enlargement of dendritic spines and decreased density of spines in FD were preserved (Haas et al., 2013). Thus, cognitive impairment in DS appears to derive from molecular and structural changes related to an altered copy number within this 33-gene region.

Among the genes from this 33-gene region, *Dyrk1a* is an attractive candidate for inducing cognitive impairment phenotypes. *DYRK1A*, the mammalian ortholog of Drosophila minibrain kinase (mnb) (Tejedor et al., 1995), encodes a proline/arginine-directed serine/threonine kinase. Both in trisomic mice and in individuals with DS, brain levels of DYRK1A are increased approximately 1.5-fold, indicating that this protein is overexpressed in a gene dosage-dependent manner (Dowjat et al., 2007). Further, comparisons of mouse models having different copy numbers of *Dyrk1a* have provided important support for the hypothesized contribution of DYRK1A to cognition. We previously assessed the molecular (i.e., immunoblotting/immunohistochemistry) and behavioral (e.g., rotarod, Morris water maze, Y-maze) consequences of alterations in *Dyrk1a* dosage in mBACtgDyrk1a, Ts65Dn, Dp(16)1Yey (each with 3 gene copies), and *Dyrk1a*+/− (one functional copy) mice (Souchet et al., 2014). Increased expression of DYRK1A in mBACtgDyrk1a induced molecular alterations in synaptic plasticity pathways, particularly expression changes in GABAergic- and glutaminergic-related proteins (Souchet et al., 2014). Similar alterations were observed in models with partial trisomy of Mmu16, Ts65Dn and Dp(16)1Yey, and were reversed in the *Dyrk1a*+/− model. Further, *Dyrk1a* overexpression produced an increased number (using stereological methodology) and an increased signal intensity of neurons expressing GAD67, an enzyme that synthesizes GABA, indicating inhibition pathway alterations in three different models. Functionally, DYRK1A overexpression protected mice from PTZ-induced seizures related to GABAergic neuron plasticity. DYRK1A dosage affects pathways involved in synaptogenesis and synaptic plasticity and influences a shift in E/I balance toward inhibition. Inhibition of DYRK1A activity offers a therapeutic target for DS, but its inhibition/activation may also be relevant for psychiatric disorders with E/I balance alterations.

Many competitive inhibitors targeting the ATP binding site of DYRK1A have been described; most also inhibit secondary targets (Ogawa et al., 2010). A comparative analysis indicates that epigallocatechin gallate (EGCG), a flavanol present in green tea, appears to inhibit DYRK1A with PRAK, another serine/threonine kinase, as a secondary target (Bain et al., 2003). Interestingly, EGCG acts non-competitively at a site external to the ATP binding site (Adayev et al., 2006). We previously assessed the effect of lifelong EGCG treatment, beginning prenatally, on the phenotype of the hYACtgDyrk1a mouse model. A dose of 50 mg/kg resulted in normal memory as measured on the novel object paradigm (Guedj et al., 2009). Following our report, a pilot clinical study performed on a group of young adults with DS found that a decaffeinated green tea extract (Mega Green Tea Extract, MGTE) improved “episodic memory test” results of the patients (De la Torre et al., 2014).

However, the mechanistic basis of the effects of EGCG treatment is not clearly established at the molecular level. Therefore, in the current study we investigated the molecular effects of a commercial green tea extract, POL60, on murine mBACtgDyrk1a and Ts65Dn models, at a dose similar to the one used in our prior report. Specifically, we assessed the effects of treatment on GABA (GAD67, GAD65, VGAT) and glutamate (GLUR1, GLUR2, NR1, NR2A, VGLUT1) pathways and on short-term memory. We also studied the effects of decaffeinated MGTE, used in the pilot clinical trial, and compared them with the effects of POL60 treatment as well as with a caffeine treatment potentially interfering with the effect of EGCG. The findings of these studies offer insights applicable to potential interventions to improve E/I balance in people with DS as well as some psychiatric disorders.

## Materials and methods

### Experimental mice

Mice were housed in standard cages with access to food and water *ad libitum*, under a controlled environment (temperature = 20 ± 1°C; humidity = 60%), and with a light/dark cycle of 12 h. All experiments were conducted in accordance with the ethical standards of French and European regulations (European Communities Council Directive, 86/609/EEC). Official authorization from the French Ministry of Agriculture was granted to perform research and experiments on animals (authorization number 75–369), and the study was approved by the local ethical committee (Univ Paris-Diderot). Mice were fed a standard laboratory diet (CRM, Special Diets Services, Dietex, France Usine). Number of mice and suffering were minimized as possible. Ts65Dn mice (Davisson et al., 1993) were maintained on a B6/C3H background and genotyped as described previously (Reinholdt et al., 2011). Mice carrying the murine BAC containing one copy of *Dyrk1A* (mBACtgDyrk1a) were maintained on a C57BL/6J background and genotyped as described (Guedj et al., 2012). (See Supplementary Table 1).

### EGCG treatment

For EGCG treatment, a final concentration of 225 mg/kg/day of Polyphenon 60 (POL60, Sigma) in water was delivered via drinking water to adult (3–4 months) male mice for 4 weeks for mBACtgDyrk1a, or for 4 weeks before and during behavioral analysis (6 weeks) for Ts65Dn; mice were euthanized at the end of treatment. Ts65Dn mice were euthanized at 6 months of age for molecular studies. POL60 contains green tea polyphenols with 27% EGCG, 42% other catechins (EC, ECG, EGC, and GC) with no effect on DYRK1A activity, and 8% caffeine; 1% sucrose was added. The placebo consisted of 1% sucrose in water. Both supplements were prepared fresh daily and offered *ad libitum*; water intake was measured on 5 days, and no difference was observed between the two groups. Decaffeinated MGTE (Life Extension) contains 45% EGCG and 53% other catechins. Solid food pellets containing MGTE were produced at a dose corresponding to 60 mg/kg/day EGCG; placebo was the ordinary solid diet. Caffeine-containing food pellets were produced at a dose corresponding to that absorbed from the POL60 supplement, i.e., 18 mg/kg/day. For each experiment, four groups of animals were used: wild-type (WT) and transgenic [TG; or trisomic (TS)] with placebo, WT and TG (or TS) with treatment.

### Behavioral analyses

To assess working memory impairment spontaneous alternation behavior was recorded in the Y-maze paradigm for the four groups of male WT and Tg/Ts animals as described in the Supplemental Methods section.

### Tissue collection

Male mice (3–4 months old for mBACtgDyrk1a were euthanized by decapitation, and brain tissue was rapidly removed. For immunoblotting, tissue was cooled on an ice block, dissected in less than 3 min, and snap-frozen in liquid nitrogen.

### Immunoblotting

Immunoblotting was performed following standard Western or slot blot protocols. Antibodies were selected by Western blot for their suitability to slot blot analyses (Guedj et al., 2012; Souchet et al., 2014) (Supplementary Table 2). Digitized images of immunoblots were obtained using a LAS-3000 imaging system (Fuji Photo Film Co. Ltd.), and densitometry measurements were collected with an image analyzer (UnScan It software, Silk Scientific Inc.). Quantification of total proteins after Ponceau-S coloration was used as an internal control.

### Statistical analysis

For comparisons between groups analyzed by two, TG/WT, TG treated/WT, TG treated/TG, *t*-tests were performed. All graphs were plotted as mean ± SEM.

Data were considered significant when *p* ≤ 0.05: in Tables 1–**6** a color code was used with red and green for significant increase and decrease respectively. A *p*-value of 0.06–0.10 was considered to indicate a strong statistical tendency due to the small sample size: in Tables 1–**6** tendancy to an increase was coded in pink and tendancy to a decrease was coded in pale green.

**Table 1:** **Protein levels of markers of inhibition and excitation pathways for WT and mBACtgDyrk1a (TG) in cortex, hippocampus, and cerebellum following treatment with EGCG-containing POL60 extract**.

**Inhibitors Comparison Markers**		**POL 60 -EGCG 67.5 mg/kg**			**POL 60 -EGCG 67.5 mg/kg**			**POL 60 -EGCG 67.5 mg/kg**	
	**TG/WT**	TG^*^/WT	TG^*^/TG	**TG/WT**	TG^*^/WT	TG^*^/TG	**TG/WT**	TG^*^/WT	TG^*^/TG
	**CTX**			**HPC**			**CRB**		
DYRK1A	162.2 ± 3.3	174.3 ± 5.4	174.3 ± 5.4	199.8 ± 9.2	208.8 ± 17	208.8 ± 17	208.7 ± 18.4	145.2 ± 11.8	145.2 ± 11.8
	*p*< 0.0001	*p*< 0.0001	*p*= 0.07	*p*< 0.0001	*p*< 0.0001	*p*= 0.64	*p*< 0.0001	*p* = 0.004	*p* = 0.01
GAD67	131.9 ± 6.6	108.1 ± 7.1	108.1 ± 7.1	142.2 ± 5.4	109.8 ± 8.4	109.8 ± 8.4	155.8 ± 8.9	117.6 ± 10.8	117.6 ± 10.8
	*p* < 0.0006	*p* = 0.35	*p* = 0.02	*p* = 0.01	*p* = 0.38	*p* = 0.004	*p* = 0.003	*p* = 0.4	*p* = 0.01
GAD65	136.4 ± 4	112.0 ± 5.1	112.0 ± 5.1	121.4 ± 2	112.7 ± 2.6	112.7 ± 2.6	145.8 ± 9.0	147.3 ± 5.4	147.3 ± 5.4
	*p* = 0.0001	*p* = 0.07	*p* = 0.0015	*p* < 0.0001	*p* = 0.001	*p* = 0.01	*p* = 0.001	*p* < 0.0001	*p* = 0.8
VGAT	123.6 ± 2.9	105.3 ± 4	105.3 ± 4	125.3 ± 4.4	99.46 ± 9.2	99.46 ± 9.2	115.5 ± 3.5	106.2 ± 2.6	106.2 ± 2.6
	*p* < 0.0001	*p* = 0.21	*p* = 0.002	*p* = 0.0003	*p* = 0.9	*p* = 0.01	*p* = 0.0004	*p* = 0.07	*p* = 0.05
GLUR1	89.70 ± 2.9	105.8 ± 4.9	105.8 ± 4.9	93.71 ± 2.4	93.44 ± 3.5	93.44 ± 3.5	67.25 ± 7.0	66.55 ± 4.09	66.55 ± 4.09
	*p* = 0.01	*p* = 0.35	*p* = 0.01	*p* = 0.1	*p* = 0.15	*p* = 0.9	*p* = 0.001	*p* < 0.0001	*p* = 0.9
GLUR2	94.93 ± 2	86.89 ± 2.6	86.89 ± 2.6	83.63 ± 1.9	95.20 ± 2.7	95.20 ± 2.7	63.61 ± 2.4	86.44 ± 3.1	86.44 ± 3.1
	*p* = 0.12	*p* = 0.001	*p* = 0.02	*p* = 0.0001	*p* = 0.23	*p* = 0.002	*p* < 0.0001	*p* = 0.01	*p* < 0.0001
NR1	92.60 ± 3	92.16 ± 5.4	92.16 ± 5.4	88.38 ± 2.2	107.8 ± 7.2	107.8 ± 7.2	96.90 ± 3.7	94.35 ± 2.1	94.35 ± 2.1
	*p* = 0.09	*p* = 0.2	*p* = 0.9	*p* = 0.02	*p* = 0.33	*p* = 0.01	*p* = 0.7	*p* = 0.45	*p* = 0.55
NR2A	87.47 ± 2.1	101.4 ± 9.3	101.4 ± 9.3	81.10 ± 1.9	100.4 ± 7.0	100.4 ± 7.0	72.12 ± 4.7	63.69 ± 3.4	63.69 ± 3.4
	*p* = 0.001	*p* = 0.9	*p* = 0.1	*p* = 0.001	*p* = 0.9	*p* = 0.01	*p* = 0.002	*p* = 0.0001	*p* = 0.17
VGLUT1	84.08 ± 1.8	107.8 ± 4.4	107.8 ± 4.4	106.3 ± 1.9	106.3 ± 2.8	106.3 ± 2.8	81.10 ± 4.5	78.04 ± 4.6	78.04 ± 4.6
	*p* = 0.0001	*p* = 0.17	*p* = 0.0001	*p* = 0.04	*p* = 0.1	*p* = 0.9	*p* = 0.01	*p* = 0.005	*p* = 0.6
VGAT/VGLUT1	149.7 ± 4.5	98.33 ± 7.3	98.33 ± 7.3	115.5 ± 4.8	92.84 ± 7.4	92.84 ± 7.4	136.7 ± 9.8	142.7 ± 9.3	142.7 ± 9.3
	*p* < 0.0001	*p* = 0.75	*p* = 0.0001	*p* = 0.02	*p* = 0.36	*p* = 0.02	*p* = 0.008	*p* = 0.002	*p* = 0.6
pCAMKII/CAMKII	86.32 ± 2.8	72.58 ± 4.5	72.58 ± 4.5	79.32 ± 3.4	99.12 ± 8.1	99.12 ± 8.1	89.30 ± 5.5	93.70 ± 3.8	93.70 ± 3.8
	*p* = 0.02	*p* = 0.0005	*p* = 0.02	*p* = 0.0004	*p* = 0.9	*p* = 0.04	*p* = 0.08	*p* = 0.5	*p* < = 0.1

Protein levels relative to the levels detected in wild-type animals (WT: 100). Mean expression relative to WT level ± standard error of the mean (SEM), P-values for the t-test comparing transgenic (TG) vs. wild-type (WT) with treated transgenic (TG^*^). (n = 10 for each genotype and for each treatment) CTX, cortex; HPC, Hippocampus; CRB, cerebellum. (Red indicates an increase, green a decrease, and blue no variation; changes were labeled for tendency toward significance with p ≤ 0.1: pink for tendancy toward increase and pale green for tendancy toward decrease).

Behavioral analyses were performed using the Mann-Whitney test as the non-normality of data precluded the use of parametric statistics (e.g., analysis of variance). All statistical analyses were performed using GraphPad6 software package.

## Results

### Effects of POL60 extract treatment

#### mBACtgDyrk1a mice

**Figure 1:**
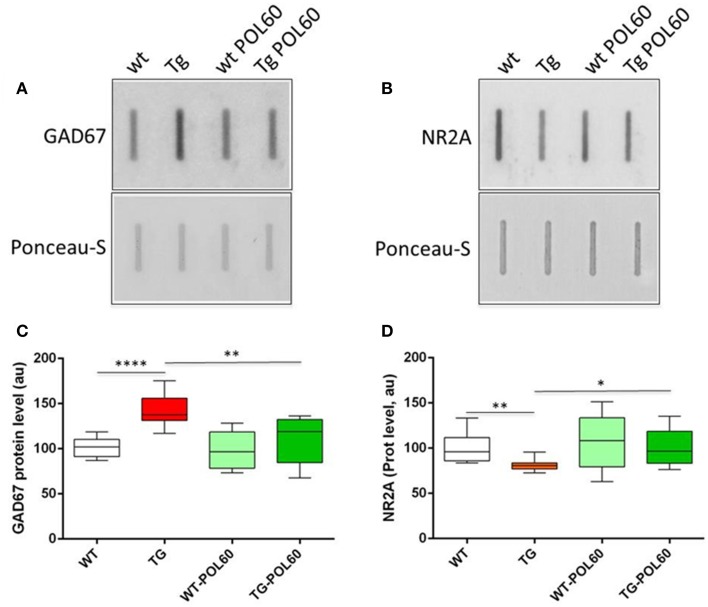
**Effect of long-term POL60 treatment on GAD67 and NR2A levels in hippocampus:** Immunoblotting of proteins from wt and mBACtgDyrk1a hippocampus treated with placebo or POL60 for **(A)** GAD67 and **(B)** NR2A. Ponceau-S coloration was used to assess total protein levels. Below: boxplots of expression relative to WT placebo (WT) for **(C)** GAD67 and **(D)** NR2A. ^*^p < 0.05, ^**^p < 0.01, ^****^p < 0.0001.

To better understand the previously observed effects of EGCG treatment on improving behavioral outcomes in DS mouse models, as well as humans with DS, adult WT and TG mice were treated for 1 month with POL60 diluted in water, with an average consumption of 3–5 mL per day, corresponding to a dose of 60 mg/kg EGCG. Markers involved in both GABAergic and glutaminergic synaptic plasticity pathways and previously characterized in various DYRK1A murine models were assessed by immunoblot to characterize the impact of treatment on E/I balance (Figure 1, Table 1). In the cortex, hippocampus, and cerebellum, overexpression of DYRK1A generally promoted higher protein levels of GABAergic markers in Tg mice compared to WT, but these levels decreased following EGCG treatment. GAD67 expression was altered in all three brain regions, while VGAT was affected only in cortex and hippocampus. In contrast, protein levels of glutaminergic markers GLUR1, NR1, NR2a, and VGLUT1 were lower in cortex of Tg mice, but their levels returned to that of WT following treatment. Similar changes were observed in the hippocampus, with the exception of VGLUT1, which was not altered in the hippocampus of transgenic animals. We observed a weaker correction of GABAergic markers in the cerebellum than in other brain regions, and no correction of glutaminergic markers in the cerebellum. The ratio of PCAMKII/CAMKII, an indicator of LTP status, was lower in TG mice in all three brain regions analyzed; a rescue effect following EGCG treatment was observed only in the hippocampus.

#### Ts65Dn mice

**Figure 2:**
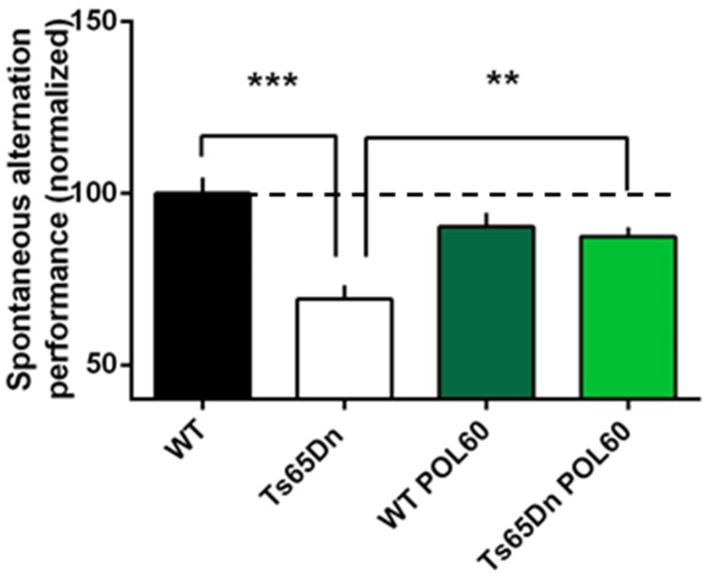
**Effect of long-term POL60 treatment on working memory in Ts65Dn mice:** Per cent of alternation was assessed in four groups of mice: WT and Ts administered placebo, *n* = 12; and WT and Ts with POL60 treatment, *n* = 8. The data have been normalized to the baseline level of performance of wild-type mice fed with water (dotted line). Ts65Dn mice displayed a very significant decrease of alternation under placebo condition. POL60 treatment produced improved alternation performance in the Ts65Dn mice. ^***^p < 0.001, ^**^p < 0.01.

The same POL60 oral treatment was applied to adult Ts65Dn animals to assess the effect of treatment in the trisomic context. After 1 month of treatment, short-term memory was assessed using spontaneous alternation in the Y maze (Figure 2). Percentage of alternation was lower in Ts65Dn animals than in WT (*P* = 0.0002). However, this difference was rescued by POL60, with a significant increase of spontaneous alternation after treatment (*p* = 0.0022).

After these behavioral assessments, mice were euthanized and brains were collected and analyzed as for mBACtgDyrk1a mice. As was observed in the TG mice, the levels of GABAergic markers were higher in all three brain regions, and levels of glutaminergic markers were lower (with the exception of GLUR1 and GLUR2 in hippocampus) in TS mice compared to WT (Table 2). However, treatment with POL60 resulted in rescued levels of GABAergic and glutaminergic markers in cortex and hippocampus. Further, the ratio of pCAMKII/CAMKII, which was significantly lower in Ts65Dn mice, was rescued by treatment. In contrast, treatment did not modify the alterations observed in the cerebellum (Table 2).

**Table 2:** **Protein levels of markers of inhibition and excitation pathways for WT and Ts65Dn (TS) in cortex, hippocampus, and cerebellum following treatment with POL60 extract**.

**Inhibitors Comparison Markers**		**POL 60 -EGCG 67.5 mg/kg**			**POL 60 -EGCG 67.5 mg/kg**			**POL 60 -EGCG 67.5 mg/kg**	
	**TS/WT**	TS^*^/WT	TS^*^/TS	**TS/WT**	TS^*^/WT	TS^*^/TS	**TS/WT**	TS^*^/WT	TS^*^/TS
	**CTX**			**HPC**			**CRB**		
DYRK1A	164.3 ± 20.3	128.7 ± 10.9	128.7 ± 10.9	168.0 ± 24.2	136.1 ± 10.9	136.1 ± 10.9	168.0 ± 24.2	136.1 ± 10.9	136.1 ± 10.9
	*p* = 0.01	*p* = 0.05	*p* = 0.12	*p* = 0.02	*p* = 0.03	*p* = 0.2	*p* = 0.02	*p* = 0.03	*p* = 0.21
GAD67	144.4 ± 20	84.34 ± 10.4	84.34 ± 10.4	189.3 ± 48.4	109.0 ± 17.4	109.0 ± 17.4	152.2 ± 22.4	147.9 ± 22	147.9 ± 22
	*p* = 0.04	*p* = 0.35	*p* = 0.01	*p* = 0.09	*p* = 0.73	*p* = 0.1	*p* = 0.06	*p* = 0.1	*p* = 0.89
GAD65	117.7 ± 7.5	105.1 ± 5.2	105.1 ± 5.2	125.8 ± 14.7	101.8 ± 4.8	101.8 ± 4.8	123.1 ± 10	128.1 ± 10	128.1 ± 10
	*p* = 0.06	*p* = 0.45	*p* = 0.21	*p* = 0.09	*p* = 0.57	*p* = 0.1	*p* = 0.09	*p* = 0.05	*p* = 0.7
VGAT	213.3 ± 42.9	87.47 ± 24.6	87.47 ± 24.6	129.1 ± 13.7	104.7 ± 5.8	104.7 ± 5.8	144.3 ± 11.8	121.6 ± 5	121.6 ± 5
	*p* = 0.05	*p* = 0.74	*p* = 0.01	*p* = 0.09	*p* = 0.68	*p* = 0.1	*p* = 0.008	*p* = 0.03	*p* = 0.08
GLUR1	84.17 ± 3.2	104.6 ± 6.0	104.6 ± 6.0	105.8 ± 5.7	98.69 ± 6.2	98.69 ± 6.2	86.66 ± 13.5	79.61 ± 8.2	79.61 ± 8.2
	*p* = 0.005	*p* = 0.53	*p* = 0.02	*p* = 0.42	*p* = 0.8	*p* = 0.4	*p* = 0.4	*p* = 0.12	*p* = 0.6
GLUR2	77.05 ± 8.04	97.08 ± 6.3	97.08 ± 6.3	106.4 ± 7.9	107.5 ± 6.8	107.5 ± 6.8	98.93 ± 9.5	98.18 ± 6.1	98.18 ± 6.1
	*p* = 0.04	*p* = 0.74	*p* = 0.06	*p* = 0.52	*p* = 0.43	*p* = 0.92	*p* = 0.94	*p* = 0.8	*p* = 0.9
NR1	105.2 ± 5.5	100.1 ± 4	100.1 ± 4	85.25 ± 3.6	96.9 ± 4.7	96.9 ± 4.7	85.25 ± 3.6	96.95 ± 4.7	96.95 ± 4.7
	*p* = 0.45	*p* = 0.94	*p* = 0.45	*p* = 0.07	*p* = 0.7	*p* = 0.08	*p* = 0.07	*p* = 0.7	*p* = 0.08
NR2A	99.87 ± 3.1	106.9 ± 3.6	106.9 ± 3.6	99.44 ± 8.1	85.09 ± 7.4	85.09 ± 7.4	82.71 ± 3.1	82.48 ± 4.4	82.48 ± 4.4
	*p* = 0.98	*p* = 0.27	*p* = 0.17	*p* = 0.7	*p* = 0.26	*p* = 0.21	*p* = 0.01	*p* = 0.02	*p* = 0.9
VGLUT1	88.45 ± 2.18	103.7 ± 3.7	103.7 ± 3.7	91.24 ± 2.4	100.5 ± 3.7	100.5 ± 3.7	106.1 ± 7.4	102.3 ± 5	102.3 ± 5
	*p* = 0.01	*p* = 0.5	*p* = 0.008	*p* = 0.02	*p* = 0.9	*p* = 0.08	*p* = 0.36	*p* = 0.47	*p* = 0.6
VGAT/VGLUT1	247.4 ± 55.5	87.86 ± 25.9	87.86 ± 25.9	142.5 ± 16.5	106.7 ± 4.3	106.7 ± 4.3	138.5 ± 12.1	118.9 ± 7.1	118.9 ± 7.1
	*p* = 0.04	*p* = 0.73	*p* = 0.01	*p* = 0.03	*p* = 0.44	*p* = 0.04	*p* = 0.01	*p* = 0.05	*p* = 0.18
pCAMKII/CAMKII	50.32 ± 12.6	102.1 ± 17.9	102.1 ± 17.9	59.08 ± 14.1	124.1 ± 20.1	124.1 ± 20.1	75.59 ± 5.3	66.06 ± 3.5	66.06 ± 3.5
	*p* = 0.05	*p* = 0.9	*p* = 0.05	*p* = 0.08	*p* = 0.36	*p* = 0.03	*p* = 0.05	*p* = 0.03	*p* = 0.15

Protein levels relative to the levels detected in wild-type animals (WT: 100). Mean expression relative to WT level ± standard error of the mean (SEM), P-values for the t-test comparing trisomic (TS) vs. wild-type (WT) with treated trisomic (TS^*^). (n = 10) CTX, cortex; HPC, Hippocampus; CRB, cerebellum. (Red indicates an increase, green a decrease, and blue no variation; changes were labeled for tendency toward significance with p ≤ 0.1: pink for tendancy toward increase and pale green for tendancy toward decrease).

### Treatment with MGTE in mBACtgDyrk1a mice: Dose effects and behavioral rescue

For translational purposes, we chose to continue our analyses with an extract used for food supplementation or direct consumption in humans: MGTE, which contains 45% EGCG and three other catechins. To select the right dose of EGCG, the effects of three doses were compared: a dose 6 times lower than the EGCG doses previously used (dose I = 10 mg/kg); a dose similar to the previous experiments (dose II = 60 mg/kg); and a dose 6 times higher than the intermediate dose (dose III = 360 mg/kg). WT and Tg adult (3–4 months) mice were treated with MGTE-supplemented solid food with an average consumption of 3–5 g per day. The same GABAergic and glutaminergic markers in cortex, hippocampus, and cerebellum were assessed following euthanization. To determine the true defect due to transgenesis, we used the average values of expression levels obtained for 10 WT/10 TG (3–5 experiments for each marker), and these values were compared to the values obtained for treated animals (*n* = 10 for WT and TG). In cortex (Table 3) we observed a tendency toward lower DYRK1A and GAD65 following treatment with low and intermediate doses. GAD67 levels were not modified by the low and intermediate doses, but were significantly higher after high-dose treatment. DYRK1A was significantly lower following high-dose treatment. VGAT and VGLUT1 levels were rescued by all three doses. NR1 and NR2A levels were rescued by low-dose treatment, and NR2A also by the intermediate dose. PCAMKII/CAMKII was rescued only by dose III. Thus, in cortex the intermediate dose (dose II) appeared to be the best compromise to rescue normal levels of VGAT/VGLUT1 and to avoid the increase in GAD67 and DYRK1A levels observed with dose III.

**Table 3:** **Protein levels of markers of inhibition and excitation pathways for WT and mBACtgDyrk1a (Tg) in cortex following treatment with 3 doses of MGTE extract**.

**Inhibitors Comparison Markers**		**MGTE- EGCG I- 10 mg/kg**		**MGTE- EGCG II- 60 mg/kg**		**MGTE- EGCGIII- 360 mg/kg**	
	**TG/WT**	TG^*^/WT	TG^*^/TG	TG^*^/WT	TG^*^/TG	TG^*^/WT	TG^*^/TG
**Cortex**							
DYRK1A	162.2 ± 3.3	139.1 ± 7.9	139.1 ± 7.9	148.2 ± 8.0	148.2 ± 8.0	193.4 ± 14.0	193.4 ± 14.0
	*p* < 0.0001	*p* < 0.0001	*p* = 0.01	*p* < 0.0001	*p* = 0.1	*p* < 0.0001	*p* = 0.02
GAD67	131.9 ± 6.6	133.7 ± 10.8	133.7 ± 10.8	131.2 ± 5.7	131.2 ± 5.7	159.5 ± 15.6	159.5 ± 15.6
	*p* = 0.0006	*p* = 0.0007	*p* = 0.8	*p* = 0.0003	*p* = 0.9	*p* < 0.0001	*p* = 0.08
GAD65	136.4 ± 4	114.3 ± 7.0	114.3 ± 7.0	115.2 ± 6.4	115.2 ± 6.4	131.3 ± 8.9	131.3 ± 8.9
	*p* < 0.0001	*p* = 0.04	*p* = 0.01	*p* = 0.05	*p* = 0.01	*p* = 0.002	*p* = 0.56
VGAT	123.6 ± 2.9	99.98 ± 5.3	99.98 ± 5.3	94.53 ± 3.7	94.53 ± 3.7	102.6 ± 3.7	102.6 ± 3.7
	*p* < 0.0001	*p* = 0.9	*p* = 0.0009	*p* = 0.35	*p* < 0.0001	*p* = 0.7	*p* = 0.0004
NR1	92.60 ± 3	99.53 ± 5.3	99.53 ± 5.3	91.98 ± 4.9	91.98 ± 4.9	85.50 ± 3.3	85.50 ± 3.3
	*p* = 0.09	*p* = 0.87	*p* = 0.1	*p* = 0.1	*p* = 0.9	*p* = 0.006	*p* = 0.1
NR2A	87.47 ± 2.1	101.8 ± 4.5	101.8 ± 4.5	98.00 ± 2.6	98.00 ± 2.6	90.54 ± 2.6	90.54 ± 2.6
	*p* = 0.001	*p* = 0.8	*p* = 0.007	*p* = 0.6	*p* = 0.006	*p* = 0.01	*p* = 0.37
GLUR1	89.70 ± 2.9	94.90 ± 4.9	94.90 ± 4.9	106.7 ± 4.5	106.7 ± 4.5	111.3 ± 6	111.3 ± 6
	*p* = 0.01	*p* = 0.39	*p* = 0.36	*p* = 0.25	*p* = 0.006	*p* = 0.15	*p* = 0.005
GLUR2	94.93 ± 2	91.06 ± 2.4	91.06 ± 2.4	98.26 ± 4.2	98.26 ± 4.2	88.80 ± 1.9	88.80 ± 1.9
	*p* = 0.1	*p* = 0.12	*p* = 0.24	*p* = 0.8	*p* = 0.5	*p* = 0.008	*p* = 0.05
VGLUT1	84.08 ± 1.8	96.27 ± 3.6	96.27 ± 3.6	95.16 ± 3	95.16 ± 3	101.2 ± 5.9	101.2 ± 5.9
	*p* = 0.0001	*p* = 0.34	*p* = 0.005	*p* = 0.19	*p* = 0.005	*p* = 0.8	*p* = 0.006
VGAT/VGLUT1	149.7 ± 4.5	118.7 ± 2.1	118.7 ± 2.1	110.0 ± 4.9	110.0 ± 4.9	115.8 ± 7.3	115.8 ± 7.3
	*p* < 0.0001	*p* = 0.02	*p* = 0.02	*p* = 0.22	*p* = 0.0001	*p* = 0.08	*p* = 0.01
*p*CAMKII/CAMKII	86.32 ± 2.8	80.14 ± 11.7	80.14 ± 11.7	52.77 ± 5.3	52.77 ± 5.3	102.1 ± 11.8	102.1 ± 11.8
	*p* = 0.01	*p* = 0.08	*p* = 0.48	*p* < 0.0001	*p* < 0.0001	*p* = 0.79	*p* = 0.08

Protein levels relative to the levels detected in wild-type animals (WT: 100). Mean expression relative to WT level ± standard error of the mean (SEM), P-values for the t-test comparing transgenic (TG) vs. wild-type (WT) with treated transgenic (TG^*^). (n = 10). (Red indicates an increase, green a decrease, and blue no variation; changes were labeled for tendency toward significance with p ≤ 0.1: pink for tendancy toward increase and pale green for tendancy toward decrease).

In hippocampus (Table 4), where the basal level of overexpression of DYRK1A was high, we observed lower, but not normal, DYRK1A levels after treatment of TG mice with the three doses. Neither GAD67 nor GAD65 were modified by the treatment. However, VGAT was rescued to a normal level at all three EGCG doses. Notably, the levels of three markers of the glutaminergic pathway, GLUR2, NR1, and NR2A, were corrected when using treatment II. For NR2A, this correction significantly exceeded the normal level.

**Table 4:** **Protein levels of markers of inhibition and excitation pathways for WT and mBACtgDyrk1a (TG) in hippocampus following treatment with 3 doses of MGTE extract**.

**Inhibitors Comparison Markers**		**MGTE-EGCG I- 10 mg/kg**		**MGTE-EGCG II- 60 mg/kg**		**MGTE-EGCGIII- 360 mg/kg**	
	**TG/WT**	TG^*^/WT	TG^*^/TG	TG^*^/WT	TG^*^/TG	TG^*^/WT	TG^*^/TG
**Hippocampus**							
DYRK1A	199.8 ± 9.2	164.9 ± 4.2	164.9 ± 4.2	129.5 ± 5.2	129.5 ± 5.2	164.4 ± 3.7	164.4 ± 3.7
	*p* < 0.0001	*p* < 0.0001	*p* = 0.004	*p* = 0.002	*p* = 0.0001	*p* < 0.0001	*p* = 0.008
GAD67	142.2 ± 5.4	123.5 ± 8.3	123.5 ± 8.3	123.8 ± 5.1	123.8 ± 5.1	148.4 ± 8.6	148.4 ± 8.6
	*p* < 0.0001	*p* = 0.01	*p* = 0.07	*p* = 0.001	*p* = 0.02	*p* < 0.0001	*p* = 0.46
GAD65	121.4 ± 2	122.1 ± 5.4	122.1 ± 5.4	118.2 ± 4.8	118.2 ± 4.8	117.1 ± 4.4	117.1 ± 4.4
	*p* < 0.0001	*p* < 0.0001	*p* = 0.8	*p* = 0.002	*p* = 0.7	*p* = 0.0006	*p* = 0.7
VGAT	125.3 ± 4.4	95.55 ± 7.2	95.55 ± 7.2	102.9 ± 5.3	102.9 ± 5.3	101.2 ± 5.4	101.2 ± 5.4
	*p* = 0.0003	*p* = 0.47	*p* = 0.002	*p* = 0.57	*p* = 0.005	*p* = 0.8	*p* = 0.003
GLUR1	93.71 ± 2.4	83.72 ± 2.4	83.72 ± 2.4	94.06 ± 6.0	94.06 ± 6.0	92.94 ± 2.5	92.94 ± 2.5
	*p* = 0.1	*p* = 0.003	*p* = 0.01	*p* = 0.31	*p* = 0.9	*p* = 0.24	*p* = 0.43
GLUR2	83.63 ± 1.9	90.28 ± 2.6	90.28 ± 2.6	99.42 ± 1.9	99.42 ± 1.9	90.49 ± 4.8	90.49 ± 4.8
	*p* = 0.0001	*p* = 0.1	*p* = 0.05	*p* = 0.9	*p* = 0.0001	*p* = 0.15	*p* = 0.26
NR1	88.38 ± 2.2	108.3 ± 6.9	108.3 ± 6.9	113.2 ± 7.1	113.2 ± 7.1	133.9 ± 7.2	133.9 ± 7.2
	*p* = 0.02	*p* = 0.31	*p* = 0.01	*p* = 0.1	*p* = 0.005	*p* = 0.0005	*p* < 0.0001
NR2A	81.10 ± 1.9	82.25 ± 4.4	82.25 ± 4.4	121.8 ± 6.7	121.8 ± 6.7	110.8 ± 4.6	110.8 ± 4.6
	*p* = 0.001	*p* = 0.01	*p* = 0.78	*p* = 0.02	*p* < 0.0001	*p* = 0.29	*p* < 0.0001
VGLUT1	106.4 ± 3.1	98.46 ± 5.7	98.46 ± 5.7	93.32 ± 4.3	93.32 ± 4.3	98.60 ± 5.0	98.60 ± 5.0
	*p* = 0.05	*p* = 0.61	*p* = 0.23	*p* = 0.05	*p* = 0.02	*p* = 0.7	*p* = 0.24
VGAT/VGLUT1	115.5 ± 4.8	89.13 ± 4.8	89.13 ± 4.8	111.2 ± 3.9	111.2 ± 3.9	103.8 ± 5.8	103.8 ± 5.8
	*p* = 0.02	*p* = 0.07	*p* = 0.001	*p* = 0.05	*p* = 0.5	*p* = 0.58	*p* = 0.1
*p*CAMKII/CAMKII	79.33 ± 3.3	75.63 ± 6.6	75.63 ± 6.6	99.77 ± 12.3	99.77 ± 12.3	96.93 ± 7.4	96.93 ± 7.4
	*p* = 0.0004	*p* = 0.005	*p* = 0.59	*p* = 0.89	*p* = 0.1	*p* = 0.81	*p* = 0.04

Protein levels relative to the levels detected in wild-type animals (WT: 100). Mean expression relative to WT level ± standard error of the mean (SEM), P-values for the t-test comparing transgenic (TG) vs. wild-type (WT) with treated transgenic (TG^*^). (n = 10). (Red indicates an increase, green a decrease, and blue no variation; changes were labeled for tendency toward significance with p ≤ 0.1: pink for tendancy toward increase and pale green for tendancy toward decrease).

In cerebellum (Table 5), the treatments induced significant decreases in levels of DYRK1A at doses I and II, and increases in GAD67 at doses II and III. VGAT1 was not modified by the treatment. The only rescuing effect was observed for GLUR1 and GLUR2, at doses II and III. The ratio of VGAT/VGLUT1, which is higher in untreated transgenic mice, was significantly increased by the three MGTE doses.

**Table 5:** **Protein levels of markers of inhibition and excitation pathways for WT and mBACtgDyrk1a (Tg) in cerebellum following treatment with 3 doses of MGTE extract**.

**Treatment Markers**		**MGTE-EGCG I- 10 mg/kg**		**MGTE-EGCG II- 60 mg/kg**		**MGTE-EGCGIII- 360 mg/kg**	
	**TG/WT**	TG^*^/WT	TG^*^/TG	TG^*^/WT	TG^*^/TG	TG^*^/WT	TG^*^/TG
**Cerebellum**							
DYRK1A	171.0 ± 9	136.5 ± 9.7	136.5 ± 9.7	139.4 ± 7.0	139.4 ± 7.0	155.6 ± 13.9	155.6 ± 13.9
	*p* < 0.0001	*p* = 0.003	*p* = 0.01	*p* = 0.0002	*p* = 0.01	*p* < 0.0001	*p* = 0.35
GAD67	159.8 ± 6.7	150.6 ± 13.0	150.6 ± 13.0	181.4 ± 10.9	181.4 ± 10.9	188.6 ± 17.3	188.6 ± 17.3
	*p* = 0.0001	*p* = 0.002	*p* = 0.5	*p* = 0.0001	*p* = 0.1	*p* < 0.0001	*p* = 0.1
GAD65	139.6 ± 5.1	138.1 ± 5.6	138.1 ± 5.6	148.2 ± 9.05	148.2 ± 9.05	168.2 ± 13.6	168.2 ± 13.6
	*p* = 0.0001	*p* < 0.0001	*p* = 0.85	*p* = 0.0002	*p* = 0.41	*p* = 0.0001	*p* = 0.04
VGAT	122.6 ± 3.2	136.6 ± 4.0	136.6 ± 4.0	133.6 ± 3.6	133.6 ± 3.6	135.3 ± 3.9	135.3 ± 3.9
	*p* < 0.0001	*p* < 0.0001	*p* = 0.68	*p* < 0.0001	*p* = 0.04	*p* < 0.0001	*p* = 0.02
GLUR1	82.87 ± 2.6	98.01 ± 5.3	98.01 ± 5.3	92.62 ± 5.3	92.62 ± 5.3	100.7 ± 6.2	100.7 ± 6.2
	*p* = 0.0005	*p* = 0.74	*p* = 0.01	*p* = 0.21	*p* = 0.1	*p* = 0.91	*p* = 0.01
GLUR2	87.94 ± 2.7	103.6 ± 3.6	103.6 ± 3.6	107.2 ± 2.8	107.2 ± 2.8	111.7 ± 4.7	111.7 ± 4.7
	*p* = 0.009	*p* = 0.48	*p* = 0.003	*p* = 0.09	*p* = 0.0001	*p* = 0.04	*p* = 0.0005
NR1	98.84 ± 1.6	90.71 ± 2.6	90.71 ± 2.6	94.11 ± 4.1	94.11 ± 4.1	94.18 ± 3.5	94.18 ± 3.5
	*p* = 0.7	*p* = 0.08	*p* = 0.01	*p* = 0.3	*p* = 0.28		*p* = 0.2
NR2A	85.2 ± 2.9	94.08 ± 5.6	94.08 ± 5.6	89.99 ± 3.6	89.99 ± 3.6	91.1 ± 3.8	91.15 ± 3.8
	*p* = 0.001	*p* = 0.4	*p* = 0.15	*p* = 0.04	*p* = 0.36	*p* = 0.07	*p* = 0.31
VGLUT1	84.5 ± 2	83.82 ± 2.7	83.82 ± 2.7	81.72 ± 3.0	81.72 ± 3.0	77.67 ± 2.1	77.67 ± 2.1
	*p* < 0.0001	*p* = 0.0005	*p* = 0.66	*p* = 0.0001	*p* < 0.33	*p* < 0.0001	*p* = 0.07
VGAT/VGLUT1	141.6 ± 5.1	161.1 ± 3.7	161.1 ± 3.7	162.0 ± 8.4	162.0 ± 8.4	173.1 ± 7.8	173.1 ± 7.8
	*p* < 0.0001	*p* < 0.0001	*p* = 0.008	*p* < 0.0001	*p* = 0.06	*p* < 0.0001	*p* = 0.004

Protein levels relative to the levels detected in wild-type animals (WT: 100). Mean expression relative to WT level ± standard error of the mean (SEM), P-values for the t-test comparing transgenic (TG) vs. wild-type (WT) with treated transgenic (TG^*^). (n = 10). (Red indicates an increase, green a decrease, and blue no variation; changes were labeled for tendency toward significance with p ≤ 0.1: pink for tendancy toward increase and pale green for tendancy toward decrease).

We used a spontaneous alternation paradigm (similar to the Y–maze experiment performed with POL60 treated Ts65Dn mice) to assess the effects of DYRK1A overexpression on short-term spatial working memory. We found that the exploratory activity in the Y-maze was affected by Dyrk1a overexpression: the total number of arm entries was higher in mBACtgDyrk1a mice compared with wild type animals in both conditions of treatment (placebo and MGTE, *p* = 0.012 and *p* = 0.009 respectively).

**Figure 3:**
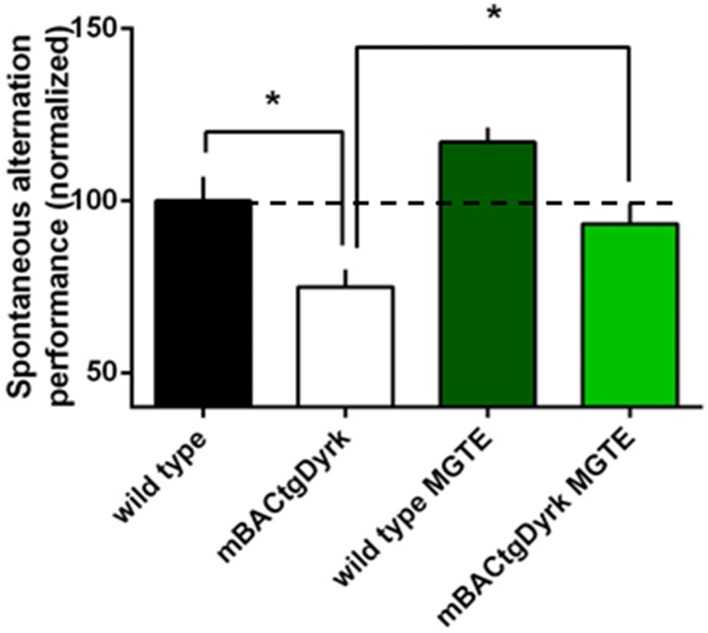
**Cognitive performance of GTE (or MGTE)-treated mBACtgDyrk1a in the Y maze**. The effects of *Dyrk1A* overexpression on short-term spatial memory were assessed in a spontaneous alternation paradigm in a Y–maze. The data have been normalized to the baseline level of performance of wild-type mice fed with water (dotted line). One mouse from the BACtgDyrk1a group was excluded from statistical analysis because of abnormally high levels of locomotor activity and associated erratic exploration, precluding assessment of memory scores. The rate of spontaneous alternation (visiting each arm in turn) was affected by genotype: mBACtgDyrk1a mice alternated less than wild-type mice (*P* < 0.05). Treatment with MGTE significantly improved performance of mBACtgDyrk1a mice. ^*^p < 0.05.

The rate of spontaneous alternation (visiting each arm in turn) was affected by genotype: mBACtgDyrk1a mice alternated less than wild-type mice (*p* = 0.017, Figure 3). Noteworthy, treatment improved the rate of spontaneous alternation of mBACtgDryk1a mice (*p* = 0.03).

### Treatment with caffeine in mBACtgDyrk1a mice: Molecular effects

To explain the differences observed in the corrections of GAD67 levels between POL60 treatment and MGTE treatment, we hypothesized an effect of the caffeine contained in green tea and present in POL60 extract. We designed a caffeine-supplemented solid diet alone with a caffeine dose (18 mg/kg) equivalent to the dose given to mice treated with POL60. After 1 month of treatment, adult mBACtgDyrk1a and wild-type mice animals were euthanized and brains collected and analyzed (Table 6). In cortex, we observed significantly decreased levels of markers of GABAergic neurotransmission: levels of GAD67 and VGAT1 and the ratio of VGAT/VGLUT1 were partially rescued. In hippocampus, rescue of these markers was complete. In contrast, in cortex and hippocampus, markers of glutaminergic neurotransmission, GLUR1 and GLUR2, remained at low levels after treatment, and the levels of NR1 and NR2A were further decreased after treatment.

**Table 6:** **Protein levels of markers of inhibition and excitation pathways for WT and mBACtgDyrk1a (Tg) in cortex and hippocampus following treatment with caffeine**.

**Inhibitors Comparison Markers**		**Caffeine**			**Caffeine**	
	**TG/WT**	TG^*^/WT	TG^*^/TG	**TG/WT**	TG^*^/WT	TG^*^/TG
	**CTX**			**HPC**		
DYRK1A	162.2 ± 3.3	180.1 ± 8.1	180.1 ± 8.1	199.8 ± 9.2	149.4 ± 14	149.4 ± 14
	*p* < 0.0003	*p* < 0.0001	*p* = 0.8	*p* < 0.0001	*p* < 0.0001	*p* = 0.006
GAD67	131.9 ± 6.6	110.3 ± 7.8	110.3 ± 7	142.2 ± 5.4	103.7 ± 9.2	103.7 ± 9.2
	*p* = 0.0006	*p* = 0.22	*p* = 0.05	*p* < 0.0001	*p* = 0.8	*p* = 0.001
VGAT	123.6 ± 2.9	109.3 ± 7.9	109.3 ± 7.9	125.3 ± 4.4	99.46 ± 9.2	99.46 ± 9.2
	*p* < 0.0001	*p* = 0.2	*p* = 0.07	*p* = 0.0003	*p* = 0.9	*p* = 0.008
GLUR1	89.70 ± 2.9	85.94 ± 2.8	85.94 ± 2.8	93.71 ± 2.4	93.44 ± 3.5	93.44 ± 3.5
	*p* = 0.01	*p* = 0.002	*p* = 0.39	*p* = 0.1	*p* = 0.1	*p* = 0.9
GLUR2	94.93 ± 2	96.86 ± 5	96.86 ± 5	83.63 ± 1.9	85.94 ± 2.8	85.94 ± 2.8
	*p* = 0.1	*p* = 0.58	*p* = 0.9	*p* = 0.0001	*p* = 0.003	*p* = 0.5
NR1	92.60 ± 3	85.18 ± 3.8	85.18 ± 3.8	88.38 ± 2.2	68.20 ± 5.6	68.20 ± 5.6
	*p* = 0.09	*p* = 0.01	*p* = 0.1	*p* = 0.02	*p* = 0.0004	*p* = 0.001
NR2A	87.47 ± 2.1	69.23 ± 4	69.23 ± 4	81.10 ± 1.9	76.56 ± 7.3	76.56 ± 7.3
	*p* = 0.001	*p* < 0.0001	*p* = 0.0005	*p* = 0.001	*p* = 0.01	*p* = 0.49
VGLUT1	84.08 ± 1.8	91.54 ± 2.7	91.54 ± 2.7	106.3 ± 1.9	98.12 ± 4.4	98.12 ± 4.4
	*p* < 0.0001	*p* = 0.01	*p* = 0.03	*p* = 0.04	*p* = 0.5	*p* = 0.15
VGAT/VGLUT1	149.7 ± 4.5	125.8 ± 9.2	125.8 ± 9.2	115.5 ± 4.8	96.09 ± 7	96.09 ± 7
	*p* < 0.0001	*p* = 0.01	*p* = 0.02	*p* = 0.02	*p* = 0.56	*p* = 0.03
pCAMKII/CAMKII	86.35 ± 3.3	100.7 ± 8.6	100.7 ± 8.6	79.32 ± 3.4	115.9 ± 5.5	115.9 ± 5.5
	*p* = 0.02	*p* = 0.9	*p* = 0.1	*p* = 0.0004	*p* = 0.01	*p* < 0.0001

Protein levels relative to the levels detected in wild-type animals (WT: 100). Mean expression relative to WT level ± standard error of the mean (SEM), P-values for the t-test comparing transgenic (TG) vs. wild-type (WT) with treated transgenic (TG^*^). (n = 10) CTX, cortex; HPC, Hippocampus; CRB, cerebellum. (Red indicates an increase, green a decrease, and blue no variation; changes were labeled for tendency toward significance with p ≤ 0.1: pink for tendancy toward increase and pale green for tendancy toward decrease).

## Discussion

We found that DYRK1A protein level is associated with expression levels of proteins involved in synaptic plasticity. Specifically, enzymes involved in decarboxylation of glutamate to produce GABA and in vesicular transport of GABA are found at higher levels in mice with three copies of *Dyrk1a*. In *Dyrk1a* single-copy mice, only GABA-producing enzymes are detected at lower levels than in WT (Souchet et al., 2014); the increase in VGAT1 in hippocampus and cortex may be compensating for these reductions. In contrast, in the cerebellum GAD67 and VGAT levels were changed in the same direction. Thus, molecular data suggest that increasing *Dyrk1a* dosage induces activation of the GABA pathway with an increased production and transport of GABA; decreasing the level of *Dyrk1a* induces a decrease in both GADs. These molecular changes offer mechanistic support for behavioral phenotypes observed in mouse models. In Ts65Dn, excessive GABAergic neurotransmission results in local over-inhibition of hippocampal circuits, which dampens hippocampal synaptic plasticity and contributes to cognitive impairments; treatment with several GABA-A receptor antagonists results in increased plasticity and improved memory deficits in Ts65Dn mice (Fernandez et al., 2007). Deficits in cognition and synaptic plasticity in Ts65Dn are also ameliorated by a selective inverse agonist of GABA-A receptor α5 subtype (Braudeau et al., 2011) or by GABA-B receptor antagonists (Kleschevnikov et al., 2012). Reducing GABA-A α5 receptor-mediated inhibition normalizes the high density of GABAergic synapse markers in the molecular layer of the hippocampus of TS mice (Martinez-Cue et al., 2013).

Here we report the phenotypic rescues observed in adult murine models of DS after a 1-month oral treatment with green tea extracts containing EGCG. We propose a molecular clue to understand the mechanisms of increased inhibition in DS and for the correcting effects of EGCG.

### EGCG-containing extracts rescue components of E/I balance

For the first time, we compared the effect of an inhibitor of DYRK1A, EGCG, on the molecular phenotypes of adult mBACtgDyrk1a mice and Ts65Dn mice. We previously showed the gene dosage effect of *Dyrk1a* on GABAergic and glutaminergic pathways in models with increased DYRK1A expression and decreased DYRK1A expression. DYRK1A dose has an impact on the levels of GABA-synthesizing enzymes GAD67 and GAD65, but also on GABA transporter VGAT. DYRK1A dose also affects excitatory processes and modifies levels of glutamate receptors GLUR1 and GLUR2, of a glutamate transporter VGLUT1, and components of the NMDA receptor, NR1 and NR2A. Further, overexpression of DYRK1A reduces the activation of CAMKII, which is accompanied by anomalous NMDAR-mediated long-term potentiation (Thomazeau et al., 2014). Treatment with POL60 (27% EGCG) and MGTE (45% EGCG), given at EGCG equivalent doses (60 mg/kg) produced corrections in the levels of most of these markers, conducive to a rescue of E/I balance in agreement with the rescue of working memory. Even if we observed some differences in molecular alterations between mBACtgDyrk1a and Ts65Dn mice, which might be linked to the additional gene context in Ts65Dn, alterations were in the same direction and were corrected by POL60 treatment in similar ways in both models.

### Molecular effects are brain-region dependent

Most of the E/I markers varied in the same direction between WT and transgenic or WT and trisomic mice when different brain regions were compared. However, in mBACtgDyrk1a VGLUT1 was decreased in cortex and cerebellum but slightly increased in hippocampus; in Ts65Dn, the same marker was decreased in cortex and hippocampus and showed a non-significant increase in cerebellum. POL60 treatment corrected these alterations in both models, with the exception of GLUR2 and pCAMKII/CAMKII in cortex and in hippocampus of mBACtgDyrk1a. In cerebellum, in mBACtgDyrk1a we observed only partial correction for GAD67 and VGAT, and in Ts65Dn we observed only a partial correction for VGAT. This difference for cerebellum is intriguing and might be due to a reduced accessibility of the drug although this hypothesis is not compatible with the effect of MGTE on DYRK1A or GLUR1-GLUR2 levels; therefore these differences are most probably due to the presence of different regulatory mechanisms in cerebellum.

### EGCG molecular effects are dose-dependent

To further analyze molecular effects of EGCG treatment we compared three doses of decaffeinated MGTE compound already used in previous mouse studies and in a pilot trial; the intermediate EGCG dose was similar to the dose used for the POL60 study. In cortex the effect of the lower and intermediate doses on DYRK1A and markers from the GABA system were similar, with a partial decrease for DYRK1A and GAD65 and a complete correction for VGAT. At the highest dose, opposite effects were observed for DYRK1A, accompanied by increased GAD67, potentially exacerbating E/I imbalance. In hippocampus, low and intermediate doses induced a partial rescue of DYRK1A and GAD67. Interestingly, the stability of DYRK1A has been associated with autophosphorylation (Himpel et al., 2001), an activity that might be decreased in the presence of inhibitors. In cerebellum, the intermediate and high doses induced increased GAD67 and ratio of VGAT1/VGLUT1. For the glutaminergic pathway in cortex, corrections NR1, NR2A, and VGLUT1 were already present for the lower dose. These findings suggest that a dose below or close to the intermediate dose is the best choice for further studies.

### EGCG molecular effects are extract-dependent and differences are explained by the presence of caffeine in POL60 extracts

We observed, particularly in cortex, that a low or intermediate dose of MGTE does not change the level of GAD67, despite the rescue of GAD67 levels following treatment with POL60 at an equivalent dose of EGCG. Comparison of composition of POL60 and decaffeinated MGTE extracts indicates that POL60 contains a dose corresponding to an 18 mg/kg caffeine diet. Therefore, hypothesizing that caffeine partially mediates the effects of POL60, we fed mBACtgDyrk1a adult mice an 18 mg/kg caffeine diet in solid food. Brain synaptic marker analysis revealed that this dose of caffeine was sufficient to induce a partial rescue of GAD67 and VGAT levels in cortex, and a complete rescue of these markers in hippocampus. Glutaminergic markers were not rescued by this treatment. The mode of action of caffeine is unknown, but might involve an effect on GAD67 via A2A receptors (Carta et al., 2002). Caffeine has no effect on glutaminergic markers; however, it rescues alterations of pCAMKII/CAMKII ratio in cortex and induces an increase in hippocampus in comparison with WT.

### EGCG-containing extracts rescue short-term memory in transgenic and trisomic models

We previously reported that EGCG treatment can rescue spatial learning (De la Torre et al., 2014) and object recognition memory (Guedj et al., 2009; De la Torre et al., 2014) deficits in Ts65Dn mice. Here, we concentrated on short-term memory impairment. In this case the cortical regions are essential to the temporary storage and the recall of information over short time periods, a general process known as working memory. Lesion experiments have shown that the prelimbic area is critically involved in working memory (Granon et al., 1994). Working memory is impaired in DS (Lanfranchi et al., 2014). Ts65Dn and Ts1Rhr models of DS with partial trisomy of Mmu16 that includes the *Dyrk1a* gene (Belichenko et al., 2009; Faizi et al., 2011) have impaired short-term memory in the spontaneous alternation paradigm. Normalization of the *Dyrk1a* copy number in TS65Dn mice improves working memory (Garcia-Cerro et al., 2014), indicating that overexpression of DYRK1A is involved in working memory alterations. In a single-gene model like the mBACtgDyrk1a mouse, our results are consistent with the idea that mice overexpressing DYRK1A have an impaired working memory. Use of EGCG treatment either in POL60 or in decaffeinated MGTE rescued working memory in a Y-maze paradigm. MGTE treatment was assessed in a pilot human clinical trial and reversed the working memory deficit in individuals with DS (De la Torre et al., 2014). Our results on the levels of synaptic markers suggest that this rescue is linked with the effect of EGCG on E/I balance. However, molecular analyses indicate that POL60 treatment induces a stronger correction of the level of proteins involved in the GABAergic pathway than decaffeinated MGTE, an effect that appears to be mediated, in part, by the presence of caffeine in POL60. We have recently shown that inhibition of DYRK1A is acting on GABA-producing enzymes at two different levels, by controlling levels of GAD67 or GAD65 proteins, but also by controlling the activity of these enzymes: the level of pyridoxal phosphate, a coenzyme of GAD67 and GAD65, is under the control of DYRK1A (Tlili et al., 2013): therefore an EGCG treatment can modify the activity of GADs enzymes by inhibiting DYRK1A activity.

## Conclusion

Results show that EGCG treatment of adult mice reverses brain molecular alterations that disrupt E/I balance. Two different extracts are also efficient to restore working memory in a single-gene model and in a partial trisomy model of DS. DYRK1A is thus a therapeutic target for Down syndrome. The panel of proteins involved in the control of synaptic plasticity and E/I balance is potentially useful to assess consequences of other therapeutic strategies, and its use may help to understand molecular mechanisms involved in these strategies. The partial rescue of components of the GABAergic pathway observed when treating adult mice with a decaffeinated green tea extract suggest the possibility of combining two drugs such as EGCG and an inverse GABA agonist (Braudeau et al., 2011) to reach a complete rescue of GABAergic and glutamatergic pathways.

### Conflict of interest statement

The authors declare that the research was conducted in the absence of any commercial or financial relationships that could be construed as a potential conflict of interest.
