# Supplementary material for: Pharmacological correction of excitation/inhibition imbalance in Down syndrome mouse models
Source: Front Behav Neurosci. 2015 Oct 20;9:267. doi: 10.3389/fnbeh.2015.00267 (PMC4611057; doi:10.3389/fnbeh.2015.00267)
Supplement: Supplementary file 1 [file Presentation1.PDF]

# Supplementary Table I

| Supplementary Table I: Primers | | |
|---|---|---|
| Models | forward | reverse |
| mBACtgDyrk1a | TGGGCCAAGCAGTTAGGAGTTT | CCATGATTACGCCAAGCTATTTAGG |
| Ts65Dn | GTGGCAAGAGACTCAAATTCAAC | TGGCTTATTATTATCAGGGCATTT |

# Supplementary Table II

Supplementary table II

| Antibodies | manufacturer/reference | Dilution (Ac Iary) | Ac IIary | Dilution (Ac II$^{ary}$) | Exposition time (scds) |
|---|---|---|---|---|---|
| anti-**DYRK1A** | RD Systems/ AF5407 | 2000 | sheep | 10 000 | 600 |
| anti-**GAD67** | Millipore / MAB5406 | 4000 | mice | 40 000 | 600 |
| anti-**GAD65** | Millipore / MAB351 | 2000 | mice | 40 000 | 240 |
| anti-**VGAT1** | Millipore/ AB5062P | 500 | rabbit | 10 000 | 240 |
| anti-**NR1** | SantaCruz / sc-1467 | 500 | goat | 10 000 | 120 |
| anti-**NR2a** | Sigma / M264 | 1000 | rabbit | 10 000 | 120 |
| anti-**GLUR1** | Millipore / 04-855 | 500 | rabbit | 10 000 | 240 |
| anti-**GLUR2** | Santacruz / sc7610 | 500 | goat | 10 000 | 240 |
| anti-**VGLUT1** | Millipore / AB5905 | 5000 | guinea pig | 10 000 | 60 |
| nti-**pCamKII** (Thr28[illegible] | SantaCruz / sc-12886R | 2500 | rabbit | 10 000 | 60 |
| anti-**CamKII** | SantaCruz / sc-5391 | 5000 | goat | 10 000 | 240 |

**Spontaneous alternation in a Y-maze paradigm**

The Y-maze consisted of three arms (57 cm long x 17 cm wide x 35 cm high) in transparent plexiglass, and assembled at 120° angles. The Y-maze was placed 70 cm above the floor and was surrounded by visual cues (e.g., posters) outside of the maze. The room was illuminated by a desk lamp to maintain an intensity of 10 ± 3 Lux throughout the entire maze. A video camera suspended above the Y-maze was used to record arm entries.

The animal was placed at the center of the maze and allowed to move freely for a 10-min session. The number of arm entries was recorded during the first 5-min period and during the entire test. The number of alternations, which was defined as a successful entry into the three arms on overlapping triplet sets, was then calculated. The percent of successful alternations per possible alternations during the first 5-min period and during the entire test was calculated. The mean and standard error of the mean were calculated for each data group. Differences between groups were analyzed by a two-way ANOVA and a Student's t-test. Statistical significance was considered at $p < 0.05$.
